# Supplementary material for: Endoplasmic Reticulum Lumenal Indicators in Drosophila Reveal Effects of HSP-Related Mutations on Endoplasmic Reticulum Calcium Dynamics
Source: Front Neurosci. 2020 Aug 10;14:816. doi: 10.3389/fnins.2020.00816 (PMC7438849; doi:10.3389/fnins.2020.00816)
Supplement: Supplementary file 4 [file Data_Sheet_2.PDF]

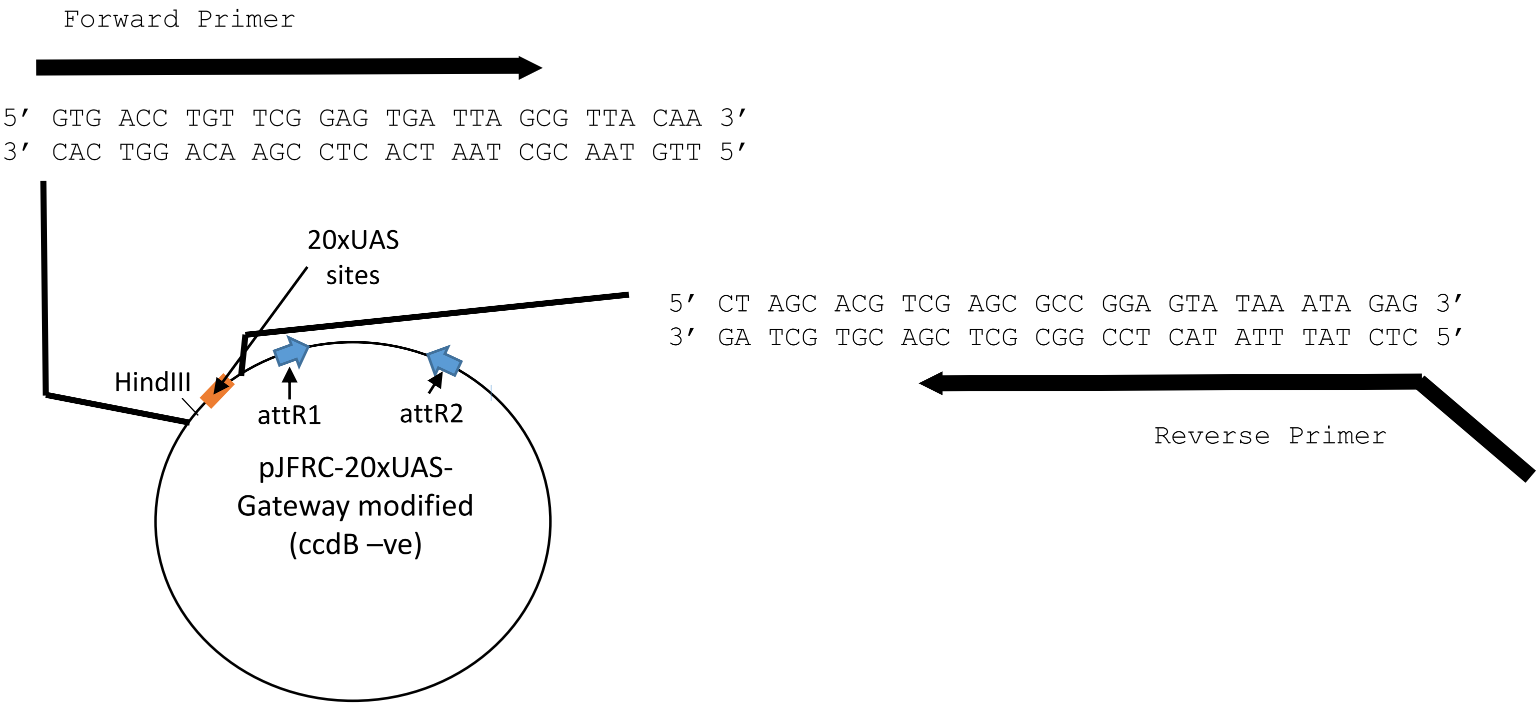


Forward Primer:

5′ GTG ACC TGT TCG GAG TGA TTA GCG 3′

Reverse Primer:

5′ AAG CTT CTC TAT TTA TAC TCC GGC GCT CG 3′
**HindIII**

PCR ↓

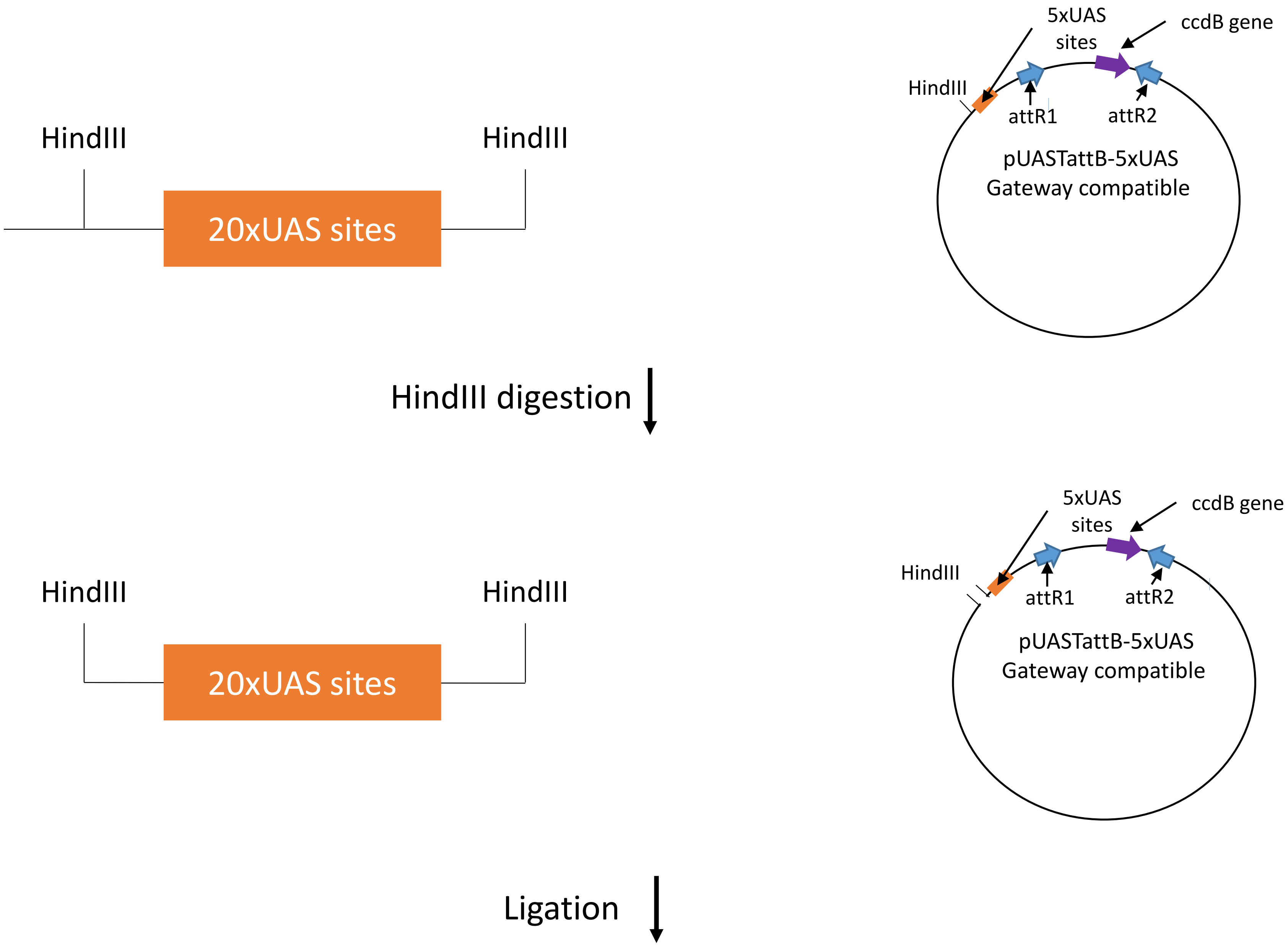
HindIII
HindIII
20xUAS sites
5xUAS sites
ccdB gene
HindIII
attR1
attR2
pUASTattB-5xUAS Gateway compatible
HindIII digestion
HindIII
HindIII
20xUAS sites
5xUAS sites
ccdB gene
HindIII
attR1
attR2
pUASTattB-5xUAS Gateway compatible
Ligation

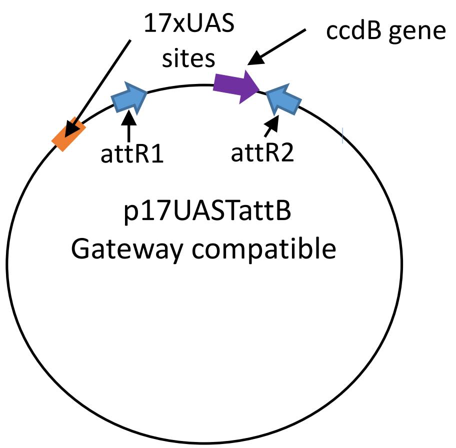
17xUAS
sites
ccdB gene
attR1
attR2
p17UASTattB
Gateway compatible

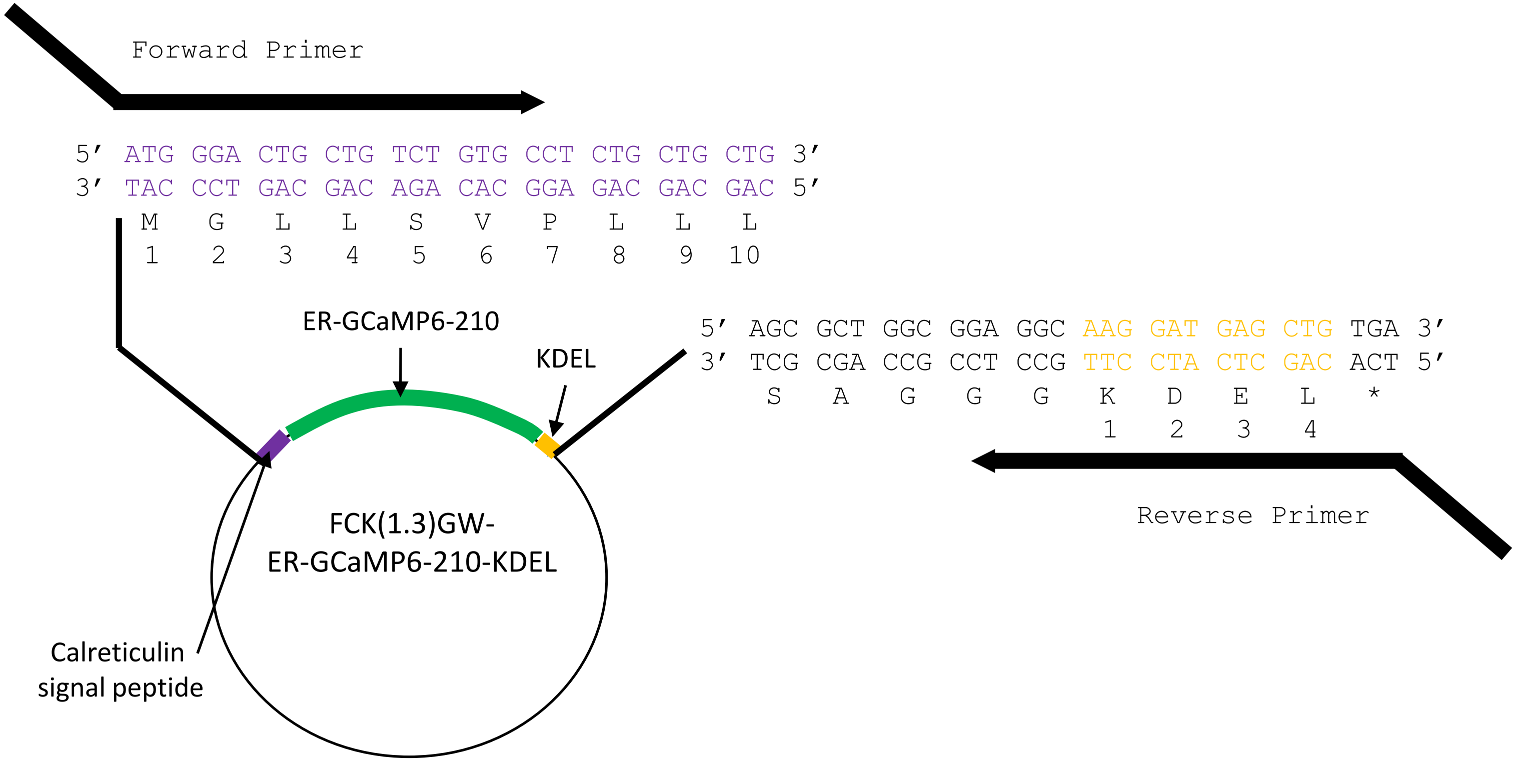


Forward Primer:

5′ GGGG ACA AGT TTG TAC AAA AAA GCA GGC TTA CAAAC ATG GGA CTG CTG TCT GTG C 3′

attB1 — GGGG ACA AGT TTG TAC AAA AAA GCA GGC TTA; **Kozak** — CAAAC

M G L L S V
1 2 3 4 5 6

Reverse Primer:

5′ GGGG AC CAC TTT GTA CAA GAA AC TGG GTT TCA CAG CTC ATC CTT GCC T 3′

attB2 — GGGG AC CAC TTT GTA CAA GAA AC TGG GTT

* L E D K G
4 3 2 1

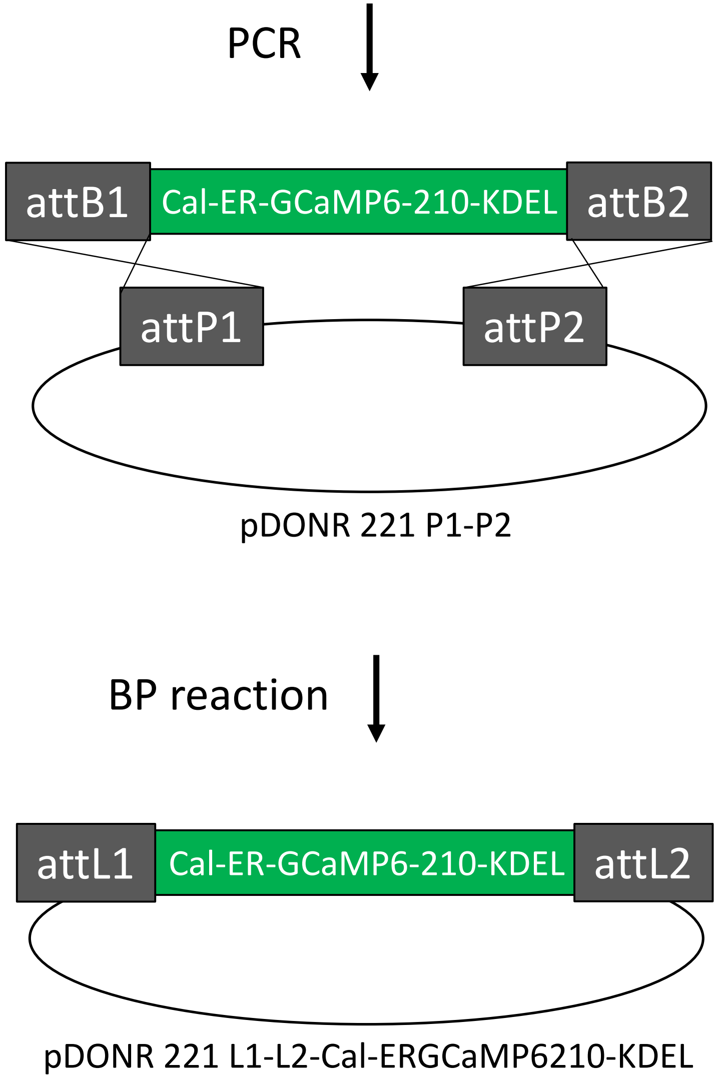
PCR
attB1
Cal-ER-GCaMP6-210-KDEL
attB2
attP1
attP2
pDONR 221 P1-P2
BP reaction
attL1
Cal-ER-GCaMP6-210-KDEL
attL2
pDONR 221 L1-L2-Cal-ERGCaMP6210-KDEL

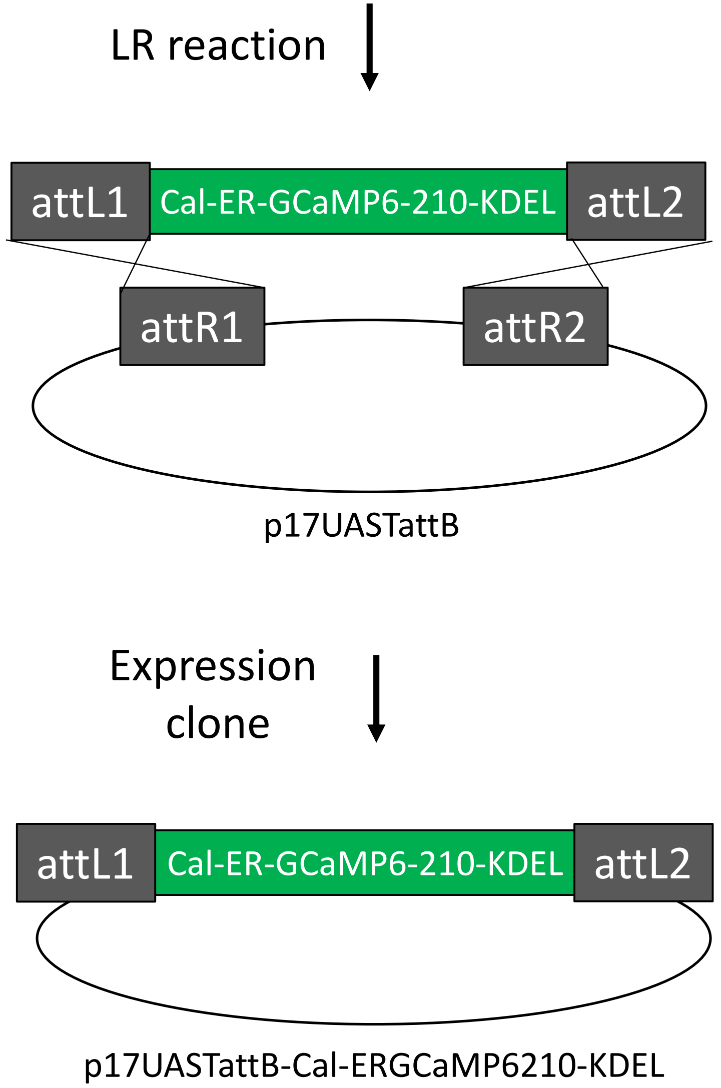
LR reaction
attL1
Cal-ER-GCaMP6-210-KDEL
attL2
attR1
attR2
p17UASTattB
Expression clone
attL1
Cal-ER-GCaMP6-210-KDEL
attL2
p17UASTattB-Cal-ERGCaMP6210-KDEL

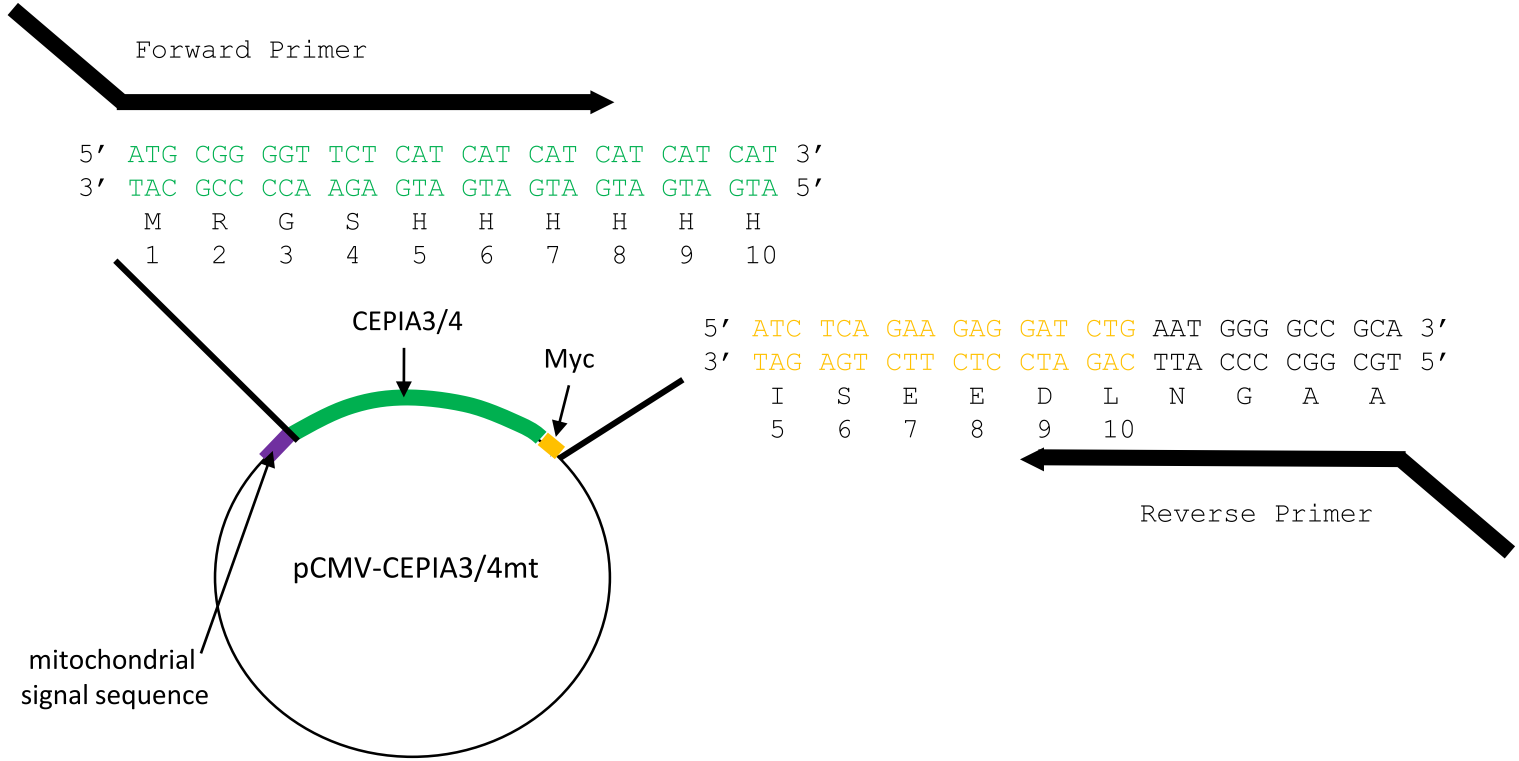


Forward Primer:

5′ GGGG ACA AGT TTG TAC AAA AAA GCA GGC TTA CAAAC ATG AAG TTA TGC ATA TTA CTG GCC GTC GTG GCC TTT GTT GGC CTC

**attB1** — **Kozak** — M K L C I L L A V V A F V G L

**BiP signal sequence**

TCG CTC GGG ACC GGT GGC GGA ATG CGG GGT TCT CAT CAT CAT C 3′

S L G T G G G M R G S H H H

1 2 3 4 5 6 7

Reverse Primer:

5′ GGGG AC CAC TTT GTA CAA GAA AC TGG GTT TCA CAA TTC GTC GTG TGC GGC CCC ATT CAG ATC 3′

**attB2** — * L E D H A A G N L D

**HDEL** — 10 9

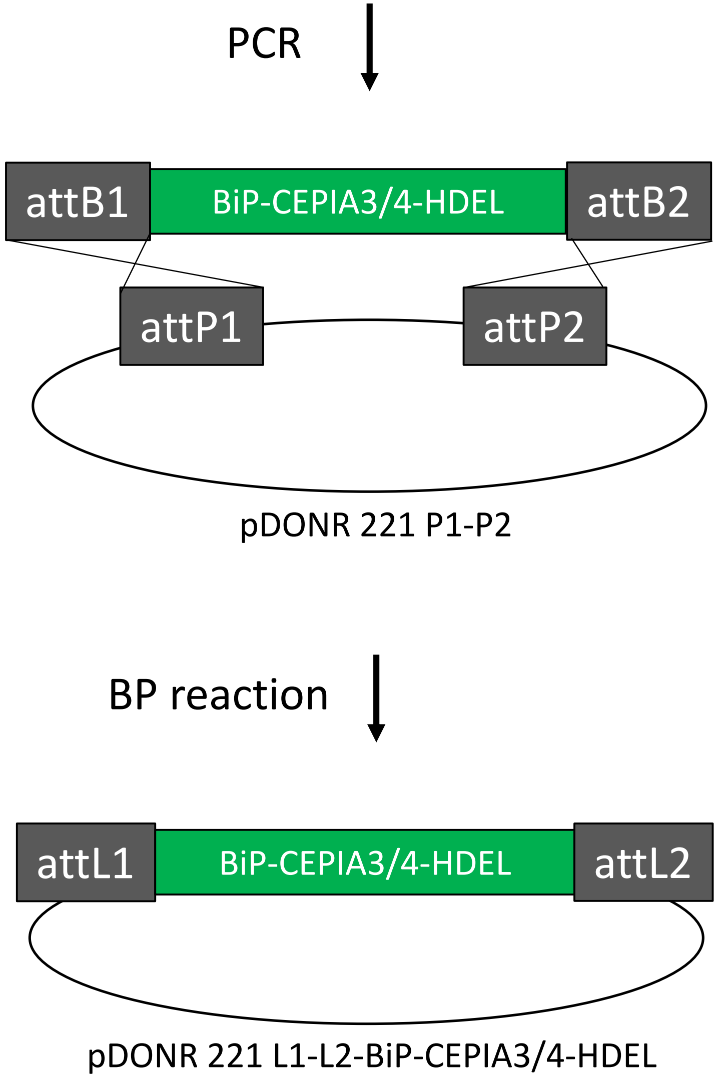
PCR
attB1
BiP-CEPIA3/4-HDEL
attB2
attP1
attP2
pDONR 221 P1-P2
BP reaction
attL1
BiP-CEPIA3/4-HDEL
attL2
pDONR 221 L1-L2-BiP-CEPIA3/4-HDEL

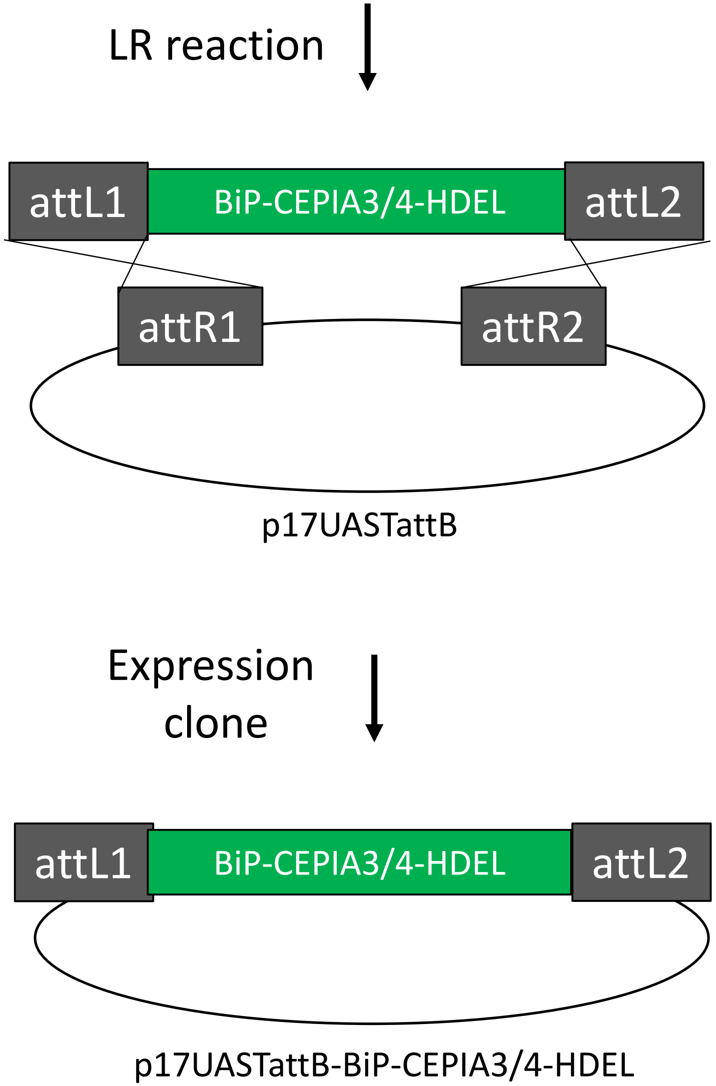
LR reaction
attL1
BiP-CEPIA3/4-HDEL
attL2
attR1
attR2
p17UASTattB
Expression clone
attL1
BiP-CEPIA3/4-HDEL
attL2
p17UASTattB-BiP-CEPIA3/4-HDEL
